# Molecular characteristics and transcriptional regulatory of spermatogenesis-related gene *RFX2* in adult Banna mini-pig inbred line (BMI)

**DOI:** 10.1590/1984-3143-AR2022-0090

**Published:** 2023-03-06

**Authors:** Zhipeng Liu, Hongmei Dai, Hailong Huo, Weizhen Li, Yun Jiang, Xia Zhang, Jinlong Huo

**Affiliations:** 1 College of Animal Science and Technology, Yunnan Agricultural University, Kunming, Yunnan, China; 2 Yunnan Vocational and Technical College of Agriculture, Kunming, Yunnan, China; 3 College of Veterinary Medicine, Yunnan Agricultural University, Kunming, Yunnan, China; 4 College of Life Science, Lyuliang University, Lvliang, Shanxi, China; 5 Department of Biology, University of Rochester, Rochester, New York, USA

**Keywords:** Banna mini-pig inbred line (BMI), whole-transcriptome sequencing, RFX2, functional annotation, transcriptional regulatory

## Abstract

*RFX2* plays critical roles in mammalian spermatogenesis and cilium maturation. Here, the testes of 12-month-old adult boars of Banna mini-pig inbred line (BMI) were subjected to whole-transcriptome sequencing. The results indicated that the average expression (raw count) of *RFX2* gene in BMI testes was 16138.25, and the average expression value of the corresponding transcript ENSSSCT00000043271.2 was 123.1898. The CDS of *RFX2* obtained from BMI testes was 2,817 bp (GenBank accession number: OL362242). Gene structure analysis showed that *RFX2* was located on chromosome 2 of the pig genome with 19 exons. Protein structure analysis indicated that RFX2 contains 728 amino acids with two conserved domains. Phylogenetic analysis revealed that RFX2 was highly conserved with evolutionary homologies among mammalian species. Other analyses, including PPI networks, KEGG, and GO, indicated that BMI RFX2 had interactions with 43 proteins involving various functions, such as in cell cycle, spermatid development, spermatid differentiation, cilium assembly, and cilium organization, etc. Correlation analysis between these proteins and the transcriptome data implied that RFX2 was significantly associated with FOXJ1, DNAH9, TMEM138, E2F7, and ATR, and particularly showed the highest correlation with ATR, demonstrating the importance of RFX2 and ART in spermatogenesis. Functional annotation implied that RFX2 was involved in 17 GO terms, including three cellular components (CC), six molecular functions (MF), and eight biological processes (BP). The analysis of miRNA-gene targeting indicated that BMI *RFX2* was mainly regulated by two miRNAs, among which four lncRNAs and five lncRNAs competitively bound ssc-miR-365-5p and ssc-miR-744 with *RFX2*, respectively. Further, the dual-luciferase report assay indicated that the ssc-miR-365-5p and ssc-miR-744 significantly reduced luciferase activity of *RFX2* 3'UTR in the 293T cells, suggesting that these two miRNAs regulated the expression of *RFX2*. Our results revealed the important role of *RFX2* in BMI spermatogenesis, making it an intriguing candidate for follow-up studies.

## Introduction

Banna mini-pig inbred (BMI) line is an important laboratory animal resource and promising donor for porcine-to-human xenotransplantation (Wang et al., 2020; Huo et al., 2022). The extreme inbreeding method, i.e., full-sibling or parent-offspring mating has been performed since 1980 in an isolated environment at Xishuangbanna, Southwest China (Huo et al., 2012). In recent years, we found that the decreased reproductivity in boars had severely restricted the population expansion of BMI (Huo et al., 2022). Therefore, investigating the molecular characteristics and transcriptional regulations of genes expressed in BMI testis will fuel future projects clarifying the expression regulation mechanism of BMI spermatogenesis.

Testes are the gonads producing sperm and hormones in male mammals (Shima et al., 2004). Spermatogenesis is a complex and developmental process regulated delicately, including the proliferation and differentiation of spermatogonium, the meiosis of spermatocyte, and the spermiogenesis process of spermatids transforming into spermatozoa (Cho et al., 2001; Pan et al., 2005). Spermatogenesis depends on the precise regulation of thousands of testis-specific genes whose defection can lead to spermatogenesis disorder or male infertility (Cho et al., 2001; Pan et al., 2005). Screening important genes related to BMI spermatogenesis and studying their expression characteristics and regulatory roles are not only crucial to uncover molecular mechanisms underlying BMI spermatogenesis disorder but also promote gene therapy to solve decreased reproductivity in BMI boars.

Cilia are microtubule organelles in vertebrates and play multiple roles in sensory reception, signal transduction, and movement. There are two major patterns in mammalian cilia, including nonmotile “9+0” primary cilia and motile “9+2” cilia, among which the sperm cilia, named flagella, is a specially motile cilium with a typical “9+2” axonemes with the nine doublet microtubules surrounding a pair of microtubules (Satir and Christensen, 2007). Cilia in mammal testis play important roles specifically at the time of postnatal development, and the formation of the flagella is essential to sperm motility and fertilization (Girardet et al., 2019). RFX2, a member of RFX2 factors, is a crucial factor of sperm flagellar assembly in mice (Emery et al., 1996a; Shawlot et al., 2015). RFX2 contains a winged-helix DNA-binding domain (DBD) that recognizes the X-Box promoter motif (Choksi et al., 2014). RFX2 is highly expressed in spermatocytes, especially round spermatids, and regulates the expression of hundreds of genes during spermatogenesis (Horvath et al., 2004). In cells, RFX2 can control the surface expansion of multi-ciliated epithelial cells (MCCs) and coordinate the expression of some genes, and regulate cell movement, ciliogenesis, and ciliary function (Chung et al., 2014). RFX2 can not only regulate ciliogenesis but also lead to left-right asymmetry through fluid flow in mice (Bisgrove et al., 2012). The deficiency of RFX2 in mouse testis leads to the difficulty of sperm flagellar production and the failure of spermatid to differentiate from round into elongated, resulting in spermatogenesis disorder and male sterility (Kistler et al., 2015; Wu et al., 2016; Yu et al., 2018). In rats, RFX2 binds to the H1t promoter, which is specifically expressed in testis and activate the transcription of *H1t* during spermatogenesis (Wolfe et al., 2006; Wolfe et al., 2008).

In this study, we performed transcriptome sequencing to obtain the expression level of *RFX2* mRNA, miRNAs, and lncRNAs of BMI testes, and carried out RT-PCR for amplifying the full-length coding sequence of *RFX2*. We analyzed the molecular characteristics of the *RFX2* gene and corresponding protein functions, and carried out protein-protein interaction and correlation analyses. We constructed the ceRNA transcriptional regulatory network of *RFX2* via annotating the *RFX2* gene and obtained the GO terms, miRNAs, and lncRNAs that interact with *RFX2*. To further validate whether the enriched miRNAs had targeting roles on RFX2, we characterized the reliability of results using the dual-luciferase report assay. Accordingly, this study underlines the importance of the high expression of *RFX2* in BMI testis, providing a valuable resource for further investigation of potential mechanisms and functions of *RFX2* gene in the process of BMI spermatogenesis.

## Methods

### Sample collection

Three 12-month-old adult BMI boars were randomly chosen, and their testis samples were obtained by surgical castration and washed with PBS buffer. After removing the fat and fascia tissues, small aliquots were crosscut from the middle area of the parenchyma, including the seminiferous tubules and immediately immersed into liquid nitrogen and transferred to -80°C freezer for storage. All animal procedures were approved by the Research Ethics Committee of Yunnan Agricultural University (No. YNAUREC2020224), and conducted according to the guideline for care and use of laboratory animals established by the National Research Council (NRC).

### Transcriptome sequencing and expression analysis of *RFX2* gene

Total RNAs of testicular samples were extracted with oligo(dT) beads (NEB, USA #E7490S). The libraries of RNA-seq and miRNAs were constructed by Novogene (Tianjin, China) and sequenced on Illumina Hiseq 4000 and Novaseq6000 platforms, respectively. The whole RNA-seq analysis included quality control of raw data using the fastp software, filtering low-quality data, and removing adaptors, sequences with N ratio greater than 10% and with all A bases. The filtered data were aligned with the rRNA reference sequence (NCBI, 2022) using Bowtie2 (V.2.1.0), and then removed the sequences that were matched with reference sequences. The pig reference genome (Sus_scrofa.Sscrofa11.1.dna.toplevel.fa) and the annotations file (Sus_scrofa.Sscrofa11.1.102.gtf) were downloaded from Ensembl, and the genomic index was constructed using STAR (v.2.5.2a). The data removed rRNA sequences were aligned with the pig reference genome. The original expression and corrected TPM values were calculated with FeatureCounts (V.2.0.1) and Salmon (V.1.5.1), respectively. The expression of *RFX2* transcript ENSSSCT00000043271.2 in BMI was obtained and visualized using the Gviz 1.40.1 package of R. The quality control analysis of Small RNA-seq was similar to the above analysis of RNA-Seq, and then removed the reads matching with porcine rRNAs, tRNAs, snRNAs, and snoRNAs of the RFAM14.8 database (http://rfam.xfam.org/), and the remaining sequences were aligned to miRBase22.1, and miRNAs were quantified using miRdeep2 (V2.0.1.3).

### Gene amplification of *RFX2*

Two pairs of primers of the *RFX2* gene were synthesized based on the transcript sequence NSSSCT00000043271.2, including F1: GATCCGGTTCCTAATAACTGAGCA, R1: ACCAGCCTTCCAAGCTCT; F2: GAGCATCACACTGCAGGACGTCA, R2: CGCGGTGGTCACAACTGTTT). The coding region sequence of *RFX2* gene was amplified using cDNA of BMI testis, and the full-length CDS sequence was spliced using Lasegene7.0. The reaction system 25 μL: Premix Taq^TM^ 12.5 μL; 10 μM primer F and primer R 1 μL, respectively; 25 ng/μL cDNA 1 μL; H_2_O 9.5 μL. Amplification program: 95 °C 5 min; 95 °C 30 sec, 57 °C 45 sec, 72 °C 80 sec, 35 cycles; 72 °C 10 min. The products were sequenced by Kunming Tsingke Biotechnology Co., Ltd.

### Functional analysis of RFX2 protein

The complete coding region sequence of *RFX2* was obtained by splicing the amplification sequences of F1/R1 and R2/R2 using Lasergene7.1, and the open reading frame (ORF) of *RFX2* was obtained using NCBI's ORFfinder. The molecular weight (Mw), molecular formula, isoelectric point (PI), and numbers of positively and negatively charged residues of the RFX2 protein were analyzed using the ProtParam. The secondary structure, hydrophobic residues, functional site, transmembrane helix, signal peptide and tertiary structure RFX2 protein were analyzed using SOPMA, ProtScale, Prosite, TMHMM 2.0, SignalP 5.0 and I- TASSER, respectively. The phylogenetic tree of RFX2 protein was generated using MEGA-X and visualized using ITOL (V6). The conserved domain of the multi-species amino acid sequence of RFX2 protein was analyzed using WebLogo3. The protein-protein interaction analysis was carried out using String11.5, and the items with *P*<0.05 were subjected to GO and KEGG enrichment analysis using clusterProfiler4.0 package of R. The correlation between the proteins enriched above and the expression levels of *RFX2* obtained from RNA-seq was calculated using cor.test function of R.

### Transcriptional regulation analysis of *RFX2*

The GO function annotation of *RFX2* was carried out using EggNOG-mapper (V2) and Uniprot. miRNAs and lncRNAs regulating *RFX2* were obtained by analyzing RNA-seq data using miRanda 3.3 and RNAhybrid 2.1.2, and the ceRNA network of transcriptional regulation was visualized using Cytoscape 3.8.2.

### Dual-luciferase reporter assay

To verify the reliability of the miRNAs obtained above acting on the 3'UTR region of the *RFX2,* we constructed the plasmids including pMIR-REPORT Luciferase-H306, pMIR-REPORT Luciferase-RFX2 3’UTR(WT)-H22978 and pMIR-REPORT Luciferase-RFX2 3’UTR(MUT1)-H22980. The 293T cells subcultured to 70% confluence were inoculated into 96-well cell culture plates, and cultured in DMEM+10% FBS, 37°C and 5% CO_2_ for 24 h. The constructed wild-type and mutant recombinant dual-luciferase reporter vectors were co-transfected into 293T cells respectively, according to the instructions of Lipofectamine^TM^2000 kit (11668019, Invitrogen). The luciferase activity was detected by the dual-luciferase reporter assay kit (E1910,Promega) in the dark. The data were calculated using the ratio of firefly luciferase activity to the renilla luciferase activity. A two-tailed t-test was performed on 3 replicates for each experiment.

## Results

### Expression characteristics of *RFX2* gene

**Figure 1:**
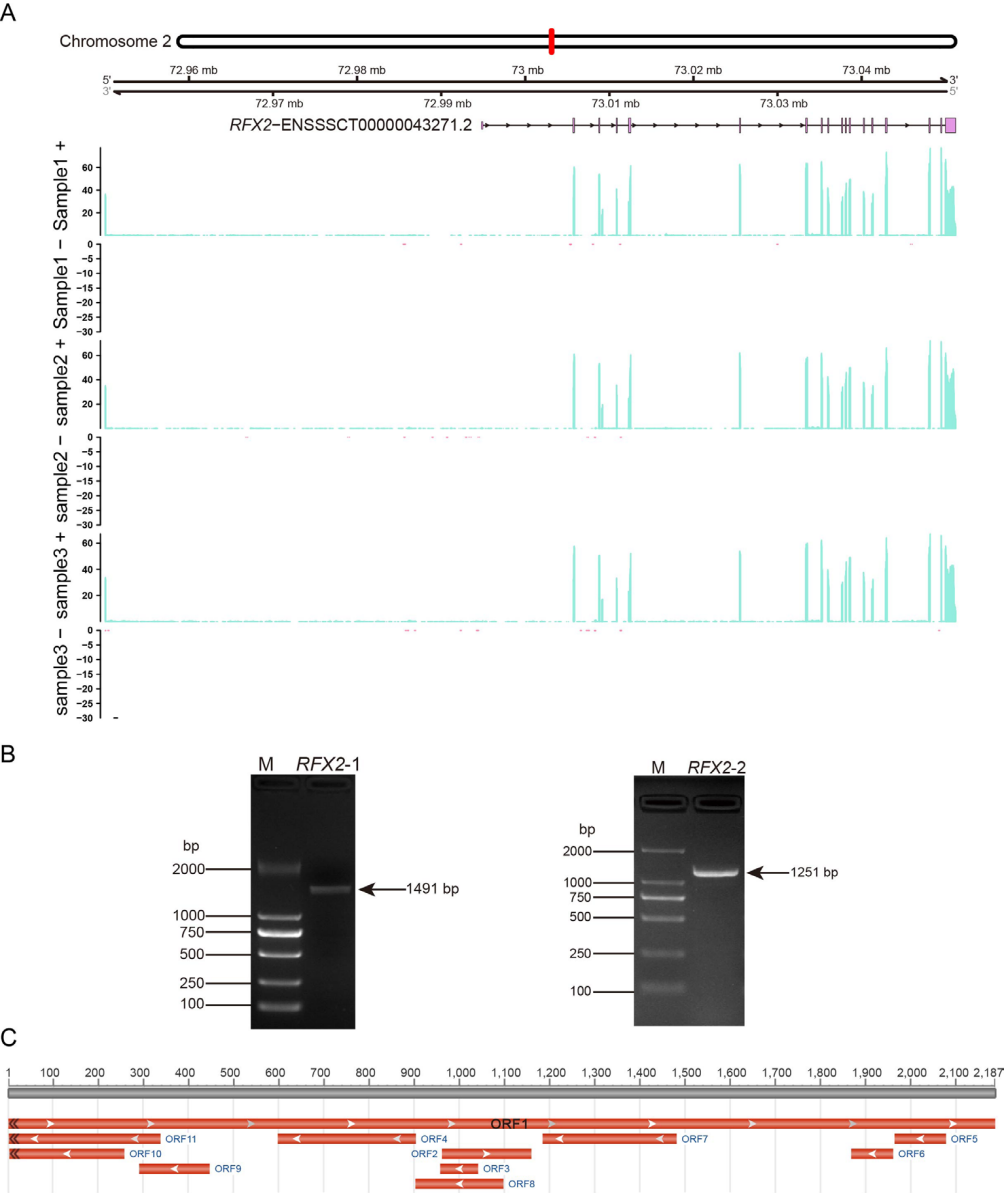
Expression analysis of *RFX2* gene and its CDS amplification and ORF acquisition. (A) chromosome location and exon, intron abundance of *RFX2* based on RNA-seq; (B) RT-PCR product of amplifying the CDS of *RFX2* gene. M. DL2000 DNA Marker. *RFX2-1* and *RFX2-1*, PCR products; (C) ORF analysis of *RFX2* cDNA.

The average expression (raw count) level of *RFX2* gene in BMI testis identified using RNA-seq was 16,138.25, and the average expression (transcripts per million, TPM) value of the corresponding transcript ENSSSCT00000043271.2 was 123.1898. *RFX2* gene was located on chromosome 2 of the pig genome Sscrofa11.1 with a full-length of 1011,26 bp. The gene annotation using Gviz 1.40.1 revealed that ENSSSCT00000043271.2 had 19 exons and 18 introns, and three BMI samples had consistently high positive expression (Figure 1A). The fragments of 1,491 bp and 1,251 bp of *RFX2* were obtained using primers F1/R1 and F2/R2, respectively (Figure 1B). Further analysis revealed that the 2,187 bp ORF1 encoding 728 amino acids was a full-length ORF (Figure 1C).

### Structural and functional analysis of RFX2 protein

The analysis of protein structure indicated the molecular weight (Mw), molecular formula and isoelectric point (PI), negative charge residues, and positive charge residues of BMI RFX2 were 80.297 kD, C_3541_H_5553_N_1007_O_1077_S_26_, 6.42, 69, 62, respectively. RFX2 protein had the largest hydrophobic value of 2.056 at position 684 and the least hydrophobic value of -3.222 at position 719, and the N-terminus and C-terminus were hydrophobic and RFX2 contained active sites such as enzymatic phosphorylation and amidation, without signal peptides or transmembrane structures.

**Figure 2:**
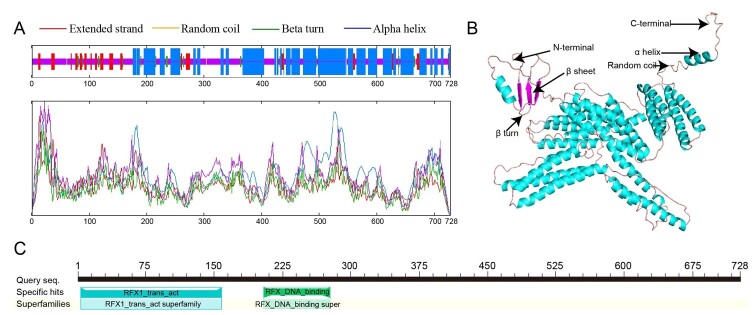
The analysis of spatial structure of RFX2 protein. (A) Secondary structure. The red, pink, green and blue vertical lines represents extended strand, random coil, beta turn and alpha helix, respectively; (B) Tertiary structure. The N-terminal, C-terminal, α helix, β sheet, β turn and random coil are indicated with black arrows; (C) The conserved domains, including RFX1_trans_act and RFX_DNA_binding.

In the secondary structure of RFX2 protein, α-helix accounted for the highest proportion with 336 amino acids for 46.15%; random coil with 302 amino acids for 41.48%; extended strand with 67 amino acids for 9.20%; β turn of the least with 23 amino acids for 3.16% (Figure 2A). Structurally, the composition of the tertiary structure of RFX2 protein was similar to that of the secondary structure, including random coil, α-helix, β sheet, β turn, and extended chain (Figure 2B). RFX2 contained two conserved domains, RFX1_trans_act and RFX_DNA_binding (Figure 2C).

### Multi-species amino acid sequence homology of RFX2

**Figure 3:**
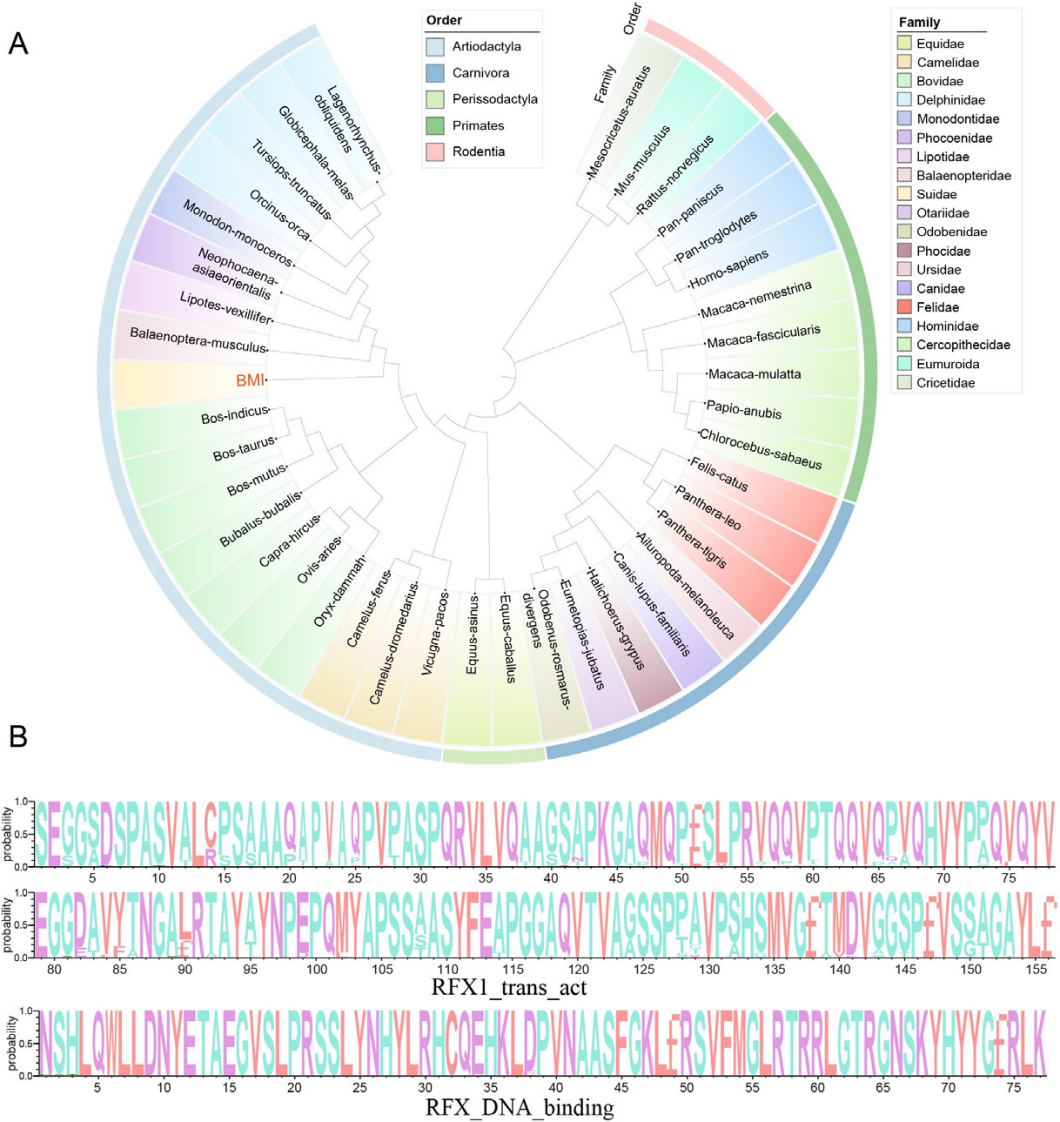
Amino acid sequences analysis of RFX2 from different mammals. (A) Phylogenetic tree for RFX2 from 40 mammalian species; (B) The analysis of conserved domains (RFX1_trans_act and RFX_DNA_binding) among 40 mammalian species using WebLogo3.

Amino acid sequence homology analysis revealed that more than 85% of RFX2 sequences in 40 mammalian species were identical. Phylogenetic analysis indicated that porcine RFX2 was grouped with Balaenoptera-musculus, Lipotes-vexillifer, etc., and then grouped with Bovidae (Figure 3A). The analysis of conserved domains of RFX2 proteins revealed that two conserved domains (RFX1_trans_act at amino acids 4-159 and RFX_DNA_binding at amino acids 202-279) were shared among 40 mammalian species. Moreover, these two conserved domains showed rather small amino acid differences among 40 species, indicating their high conservation in evolution (Figure 3B).

### Protein interaction network and gene correlation analysis

**Figure 4:**
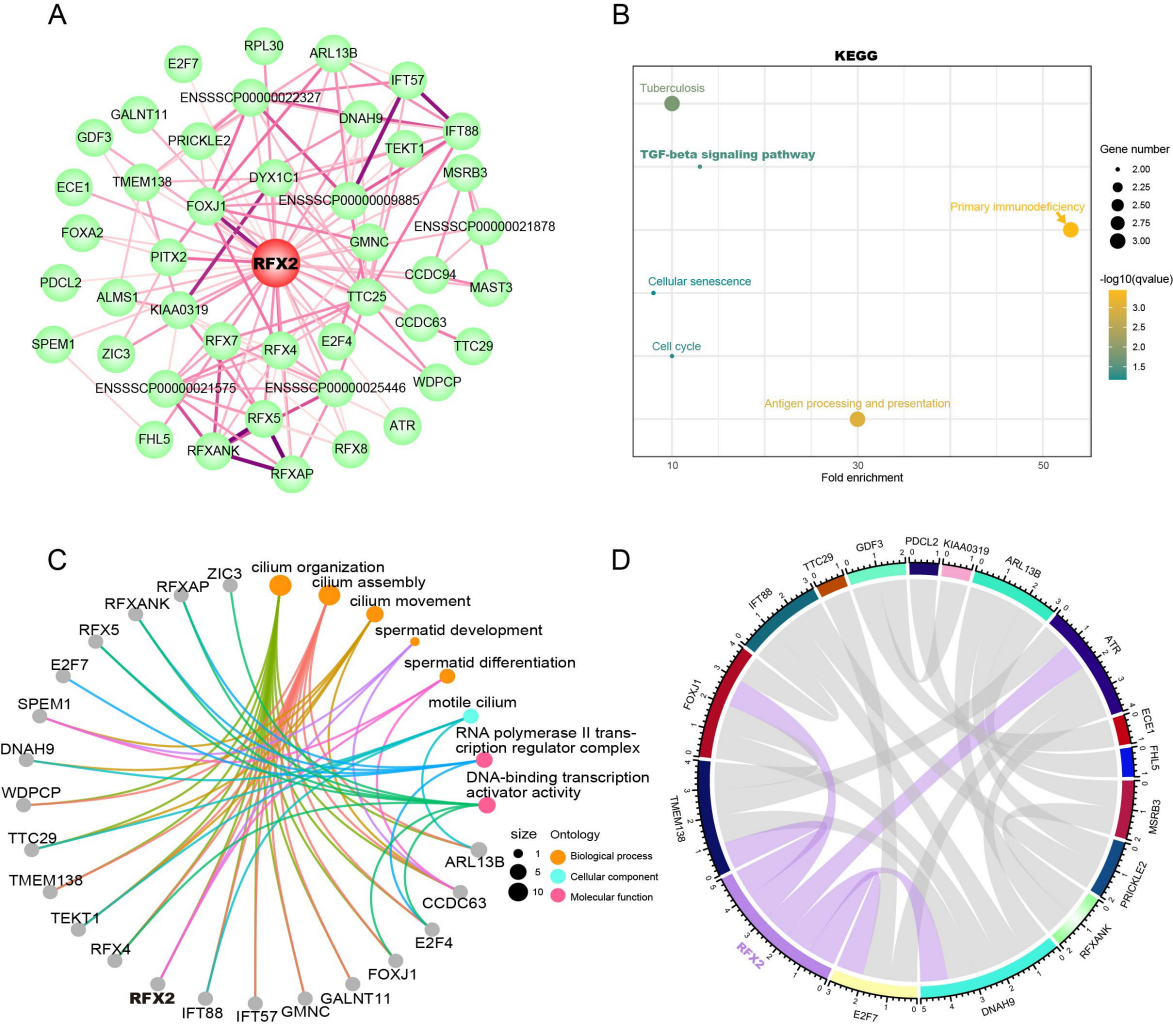
Interaction network of RFX2. (A) Protein-protein Interaction networks. The lines indicate the interactions among proteins, and more lines indicate higher confidence; (B) Interaction network of KEGG enrichment of RFX2; (C) Interaction network of GO enrichment of RFX2, including proteins and the pathways related to biological process, cellular component and molecular function; (D) Chord plot of genes correlating with the RFX2. The pink lines in the circle indicate a significant correlation with RFX2.

Protein-Protein Interaction Networks Functional Enrichment Analysis revealed potential interactions between RFX2 and 43 proteins (Figure 4A). To further investigate the functions of *RFX2*, Gene Ontology (GO) and Kyoto Encyclopedia of Genes and Genomes (KEGG) enrichment were implemented. KEGG enrichment analysis revealed that these proteins were mainly involved in cell cycle, TGF-beta signaling pathway, cellular senescence and other pathways (Figure 4B). GO enrichment revealed that these proteins were mainly involved in spermatid development, spermatid differentiation, cilium assembly and cilium organization, etc. (Figure 4C). Subsequently, we analyzed the correlation between these proteins obtained above and our RNA-seq data and constructed the expression correlation network (Figure 4D), and we found that RFX2 was significantly correlated with FOXJ1, DNAH9, TMEM138, E2F7 and ATR (Table 1).

**Table 1:** Genes that significantly correlating with the *RFX2*.

**Node1**	**Node2**	**Correlation**	**Pvalue**
*RFX2*	*FOXJ1*	0.997465	0.045336
*RFX2*	*DNAH9*	0.998620	0.033444
*RFX2*	*TMEM138*	0.998728	0.032110
*RFX2*	*E2F7*	0.999118	0.026740
*RFX2*	*ATR*	0.999916	0.008247

### ceRNA regulatory network of *RFX2*

**Figure 5:**
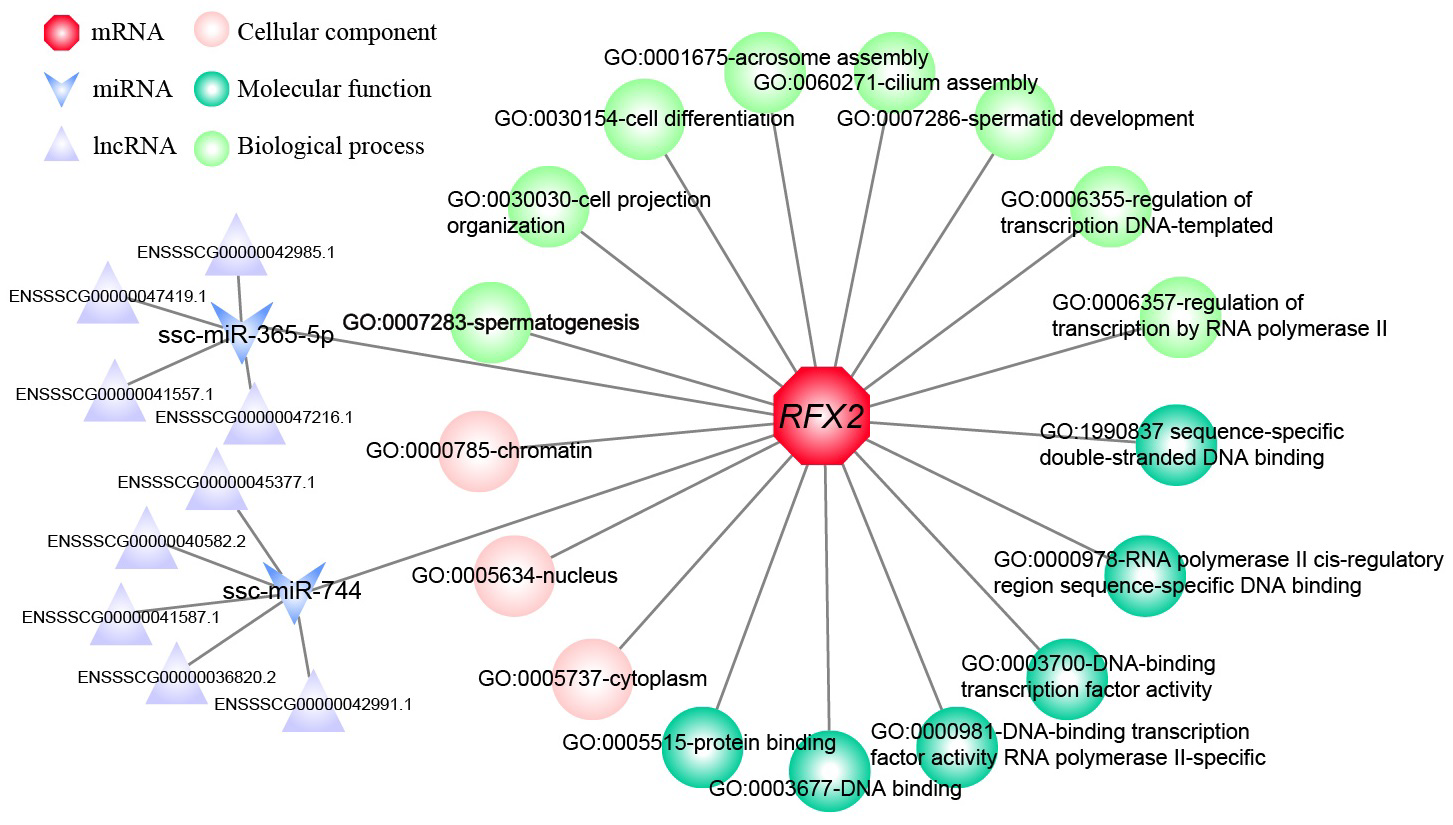
The functional annotation of BMI *RFX2* and potential ceRNA regulatory network. The hexagon, “V”, triangle, sphere, respectively represent *RFX2* mRNA, miRNA, lncRNA and GO pathways including cellular component and molecular function, biological process.

Functional annotation indicated that *RFX2* mRNA mainly involved three GO terms, including cytoplasm, nucleus, and chromatin in the Cellular Component (CC), and six GO terms including sequence-specific double-stranded DNA binding, RNA polymerase II cis-regulatory region sequence-specific DNA binding, DNA-binding transcription factor activity, DNA-binding transcription factor activity RNA polymerase II-specific, DNA binding and protein binding in the Molecular Function (MF), and eight GO terms including spermatogenesis, cell projection, cell differentiation, acrosome assembly, cilium assembly, spermatid development, regulation of transcription DNA-templated, regulation of transcription by RNA polymerase II in the Biological Process (BP) (Figure 5). *RFX2* was mainly regulated by two miRNAs, including ssc-miR-365-5p and ssc-miR-744. Of note, we found that four (ENSSSCG00000042985.1, ENSSSCG00000047419.1, ENSSSCG00000041557.1, ENSSSCG00000047216.1) and five lncRNAs (ENSSSCG00000045377.1, ENSSSCG00000040582.2, ENSSSCG00000041587.1, ENSSSCG00000036820.2, ENSSSCG00000042991.1) competitively bound ssc-mir-365-5p and ssc-mir-744 with *RFX2*, respectively (Figure 5).

### Validation of *RFX2*-miRNAs targeting relationship

**Figure 6:**
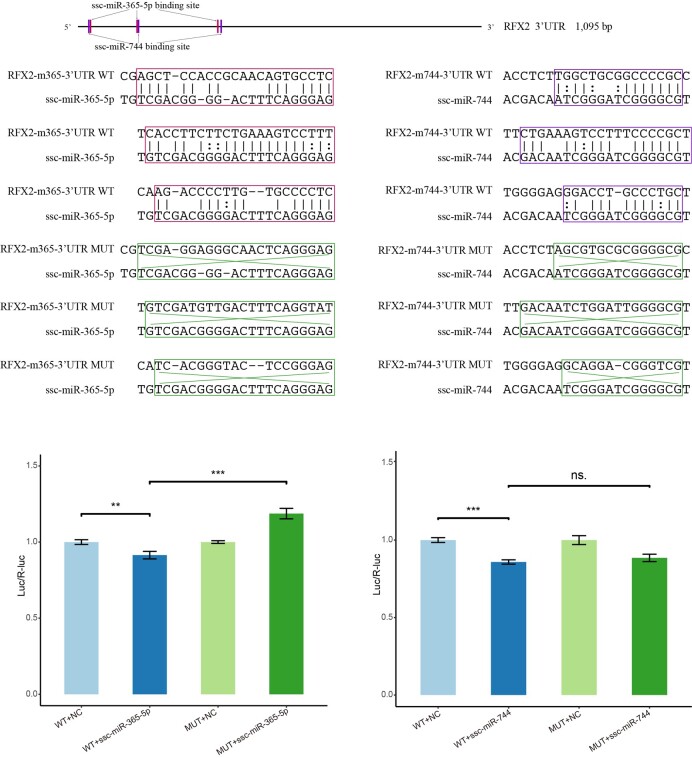
The validated results of *RFX2* regulation by miRNA ssc-miR-365-5p and ssc-miR-744. The left and right indicate ssc-miR-365-5p and ssc-miR-744, respectively. ns.: no significant difference. **, *P*<0.01. ***, *P*<0.001.

To identify the targeting effect of miRNA on *RFX2*, we verified the function of ssc-miR-365-5p and ssc-miR-744 based on the dual-luciferase reporter assay. Compared with the luciferase assay performed on the *RFX2* 3'UTR mutant reporter, the luciferase activity of 3'UTR wild-type reporter vector was significantly decreased by ssc-mir-365-5p (*P*<0.001) in 293T cells (Figure 6). Similarly, ssc-mir-744 also significantly reduced the luciferase activity of *RFX2* 3'UTR wild-type reporter vector in 293T cells (*P*<0.001), but the mutant reporter vector was not affected (*P*>0.05), suggesting that there may be other unknown binding sites of ssc-miR-744 in the *RFX2* 3'UTR. These results indicated that ssc-miR-365-5p and ssc-miR-744 did regulate the expression of *RFX2.*

## Discussion

RFX factors typically share a winged-helix DNA-binding domain (DBD), which can recognize the minor groove of promoter DNA by monomer or dimers form (Gajiwala et al., 2000). DBD is a highly conserved motif of dimeric binding protein, which has various functions in regulating unicellular organisms and eukaryotic organisms (Emery et al., 1996b; Cornille et al., 1998). RFX containing DBD motif is a regulator that can bind to MHC class II genes and regulate their expression (Nekrep et al., 2002). Domain RFX1_trans_act locates the transcriptional activation region of the N-terminal of RFX2, binds to DNA and activates transcription (Katan et al., 1997). The amino acid sequences of the two domains were slightly different among the 40 species, indicating that these two domains are highly conserved among mammals.

Protein-protein interaction analysis revealed that RFX2 interacted with 43 proteins, and KEGG enrichment analysis indicated that these proteins were involved in cell cycle, TGF-beta signaling pathway, cellular senescence, etc. GO enrichment analysis revealed that these proteins were involved in spermatid development, spermatid differentiation, cilium assembly, DNA-binding transcription activator activity, RNA polymerase II transcription regulator complex, motile cilium and cilium movement, etc. Together, these signaling pathways formed a complex network to regulate spermatogenesis and ciliogenesis. After querying the gene expression levels of these proteins in our RNA-seq data, we calculated the correlation of expression levels. Our results showed that RFX2 was significantly correlated with ATR, E2F7, TMEM138, DNAH9 and FOXJ1. ATR plays important roles in the cell-cycle and early mammalian development. Targeted disruption of ATR leads to the death of early mice embryos (de Klein et al., 2000). In humans, when DNA is damaged, the kinase encoded by ATR gene is activated as the checkpoint transducer to regulate cell cycle and DNA replication through phosphorylating a variety of substrates, enabling cell survival (Karnitz and Zou, 2015). E2F7 is a transcriptional repressor encoding cell cycle-related proteins, which inhibits cell proliferation by regulating the transcription of miRNAs (Mitxelena et al., 2016). E2F7 is essential to embryonic development and cell survival, and its dysfunction leads to apoptosis and dilation of blood vessels, resulting in embryonic death in mice (Li et al., 2008). TMEM138 is a connecting protein for cilium transport and biogenesis, and mice with mutated TMEM138 suffer from impaired primary cilia, learning and memory, resulting in Joubert syndrome (Guo et al., 2022). DNAH9 expressed on sperm tail is an important heavy chain component of the dynein arm of cilia and flagella, and mutation of DNAH9 leads to primary ciliary dyskinesia (PCD) and nonsyndromic asthenozoospermia (Fassad et al., 2018; Tang et al., 2021). FOXJ1 plays an important role in the cilia gene express by synergistically cooperating with RFX2 (Koay et al., 2021). RFX2 stabilizes the proximal promoter and distal enhancer and facilitates the core domains bound by FOXJ1 enabling the functional roles of cilia genes (Quigley and Kintner, 2017). Taken together, the findings of these proteins interacting with RFX2 will provide information and research directions for shedding further light on the function and molecular mechanism underlying the RFX2 in BMI.

Spermatogenesis is a complex biological process, which is regulated not only by mRNAs but also by ncRNAs (Gao et al., 2020). microRNAs (miRNAs) are highly conserved endogenous non-coding small RNAs with ~22 nt length that regulate gene expression post-transcriptionally (Calin and Croce, 2006; Gao et al., 2020). A growing number of reports have revealed that miRNAs are required for primordial germ cell development and spermatogenesis and play vital roles in the development of testis and spermatozoa as potential biomarkers (Hayashi et al., 2008). The functional annotation of *RFX2* and analysis of the regulatory network on competing endogenous RNA (ceRNA) indicated that *RFX2* was targeted by two miRNAs, ssc-miR-365-5p and ssc-miR-744.

miR-365-5p regulated some genes involved in modifying miRNA biogenesis (Singh and Storey, 2021), and miR-365-5p in porcine milk prevented the damage of intestinal epithelial cells via regulating the expression of p53 (Xie et al., 2020). miR-365-5p in cervical cancer was also a benign regulator that inhibited the production of Interleukin 6 (Arenas-Padilla and Mata-Haro, 2018). miR-744 was a potential prognostic marker for patients with liver cancer and inhibited the growth of Hepatocellular carcinoma (HCC) cells by targeting c-MYc (Tan et al., 2015). As tumor suppressor microRNA, miR-744 inhibited the proliferation of colorectal cancer cells by targeting RFC2 (Hu et al., 2020). miR-744-5p inhibited multiple myeloma proliferation by targeting the SOX12/Wnt/β-catenin pathway (Guo et al., 2021), and inhibited the proliferation and metastasis of HCC by targeting TGF-β1 (Huang et al., 2021). Conversely, miR-744 increased the risk of pancreatic cancer, prostate cancer and nasopharyngeal carcinoma by activating the Wnt/β-catenin pathway, AMPK signaling, and transcriptional regulation of ARHGAP5, respectively (Zhou et al., 2015; Zhang et al., 2019). The results identified by the dual-luciferase reporter assay indicated that ssc-miR-365-5p and ssc-miR-744 regulated the expression of *RFX2*, which will pave the way for further study on the mechanism of ssc-miR-365-5p and ssc-miR-744 targeting *RFX2*.

## Conclusion

In this study, RNA-seq technology was used to obtain the expression level of *RFX2* gene in the adult BMI testis and the full-length coding sequence of *RFX2* was obtained by RT-PCR. The molecular characteristics of *RFX2* gene and corresponding conserved protein domains and evolutionary relationships among multiple mammals were obtained by RNA-seq data combined with bioinformatics analysis. RFX2 was mainly involved in cell cycle, TGF-beta signaling pathway, cellular senescence and spermatid development, spermatid differentiation, cilium assembly and cilium organization, etc. RFX2 was significantly correlated with FOXJ1, DNAH9, TMEM138, E2F7 and ATR, in particular, with the strongest correlation with ATR protein. RFX2 was involved in 17 GO terms, including 3 cellular components, 6 molecular functions and 8 biological processes. The dual-luciferase reporter assay verified that *RFX2* was mainly regulated by two miRNAs, ssc-miR-365-5p and ssc-miR-744. These results broaden the understanding of transcriptional regulatory characteristics of spermatogenesis-related gene *RFX2*, thus building a foundation for further studying function and molecular mechanism of *RFX2* in BMI testis.
